# The Benefits and Challenges of Providing School Meals during the First Year of California’s Universal School Meal Policy as Reported by School Foodservice Professionals

**DOI:** 10.3390/nu16121812

**Published:** 2024-06-08

**Authors:** Monica D. Zuercher, Dania Orta-Aleman, Juliana F. W. Cohen, Christina A. Hecht, Kenneth Hecht, Michele Polacsek, Anisha I. Patel, Lorrene D. Ritchie, Wendi Gosliner

**Affiliations:** 1 Nutrition Policy Institute, Division of Agriculture and Natural Resources, University of California, Oakland, CA 94607, USA; dorta@ucanr.edu (D.O.-A.); ceahecht@ucanr.edu (C.A.H.); kenhecht@ucanr.edu (K.H.); lritchie@ucanr.edu (L.D.R.); wgosliner@ucanr.edu (W.G.); 2 Center for Health Inclusion, Research and Practice (CHIRP), Merrimack College, 315 Turnpike Street, North Andover, MA 01845, USA; jcohen@hsph.harvard.edu; 3 Department of Nutrition, Harvard T.H. Chan School of Public Health, 677 Huntington Ave., Boston, MA 02115, USA; 4 Center for Excellence in Public Health, University of New England, 716 Stevens Ave., Portland, ME 04103, USA; mpolacsek@une.edu; 5 Division of General Pediatrics, Stanford University, Palo Alto, CA 94304, USA; anipatel@stanford.edu

**Keywords:** NSLP, SBP, universal school meals, nutrition, children

## Abstract

States in the U.S. are newly implementing universal school meal (USM) policies, yet little is known about the facilitators of their success and the challenges they confront. This study evaluated the challenges and facilitators faced by school food authorities (SFAs) implementing California’s universal school meal (USM) policy during its inaugural year (2022–2023) using an online survey. In March 2023, 430 SFAs reported many benefits, including increased meal participation (64.2% of SFAs) and revenues (65.7%), reduced meal debt (41.8%) and stigma (30.9%), and improved meal quality (44.3%) and staff salaries (36.9%). Reported challenges include product/ingredient availability (80.9%), staffing shortages (77.0%), vendor/distributor logistics issues (75.9%), and administrative burden (74.9%). Top facilitators included state funding (78.2%) and increased federal reimbursement (77.2%). SFAs with fewer students eligible for free or reduced-price meals (as opposed to SFAs with more) reported greater increases in meal participation and reductions in stigma but also more administrative burdens. Larger SFAs reported greater increases in revenues, staff salaries, and improvements in meal quality than smaller SFAs but also more challenges. Overall, California’s USM policy has enhanced student access to healthy meals while mitigating social and financial barriers. Understanding California’s experience can inform other jurisdictions considering or implementing similar policies.

## 1. Introduction

The National School Lunch Program (NSLP) and the School Breakfast Program (SBP) require United States (U.S.) public schools to serve meals that meet nutritional standards established by the U.S. Department of Agriculture (USDA) Food and Nutrition Service [1,2]. Historically, children were determined eligible for free school meals if their family income was 130% of the Federal poverty level or below, through participation in certain Federal Assistance Programs (e.g., Supplemental Nutrition Assistance Program [SNAP]), or based on their status as a homeless, migrant, runaway, or foster child [1,2]. Children from families with incomes between 130 and 185% of the Federal poverty level were eligible for reduced-price meals [1,2]. Unfortunately, this tiered eligibility system resulted in barriers to school meal access for many children, including stigma around meal participation, difficulties filling out meal applications, and cutoff points to determine student eligibility that ignored factors like the cost of living [3,4]. A universal school meal (USM) program that offers school meals free of charge to all students has the potential to resolve some of these issues, while providing multiple benefits to students and their families [5,6,7].

In 2020, in response to the COVID-19 pandemic, waivers that allowed schools to offer meals free of charge to all students in school years (SYs) 2020–2022 were extended nationwide [8]. Research showed that, despite the multiple challenges faced while implementing this federally funded USM program, it provided numerous benefits to schools, students, and families, including increases in student participation in school meal programs and reductions in stigma, meal debt, financial burden, and stress for families, as well as in the administrative paperwork [4,6,9,10]. Demonstrating the impacts of this federally funded USM program helped some states pass USM legislation starting in SY 2022–2023 or in SY 2023–2024, and some additional states have introduced USM bills or formed coalitions to do so [11]. However, schools in most states returned to the previous tiered eligibility system in SY 2022–2023 [12].

In 2021, California led the USM movement in the U.S. by passing a USM policy that started in SY 2022–2023 [13]. The California USM program has three key pillars: (1) schools are required to offer breakfast and lunch for all students each school day, regardless of their free or reduced-price meals (FRPM) eligibility; (2) schools are required to optimize the federal reimbursements for which they are eligible; and (3) the California State Legislature allocates funds to cover the cost of providing meals to all students beyond federal reimbursements [13]. Moreover, the state of California made significant investments to support its USM program and to improve the quality and healthfulness of school meals, including funds to upgrade school kitchens, provide additional training and technical assistance, and support the California Farm to School Program, among others [14]. 

There are differences between USM policies funded by the federal government under Coronavirus disease (COVID-19) waivers and the current California USM program. The latter requires all schools to offer both school lunch and breakfast; high-poverty schools are required to participate in a federal provision (Community Eligibility Provision (CEP) or Provision 2 or 3), and schools are required to determine students’ FRPM eligibility for the meal claims and reimbursement, and meal reimbursement rates are higher (increased in SY 2022–2023) [13]. Also, many COVID-19 waivers that allowed schools to serve school meals outside of the previous tiered approach had expired by the time the California USM program started in the SY 2022–2023 [8,15]. In addition to state investments, multiple federal investments have been made in recent years to improve school meal programs, including funds to purchase kitchen equipment, train foodservice staff, and improve access to local foods [14,16]. Moreover, the social and political environment has evolved over time, and while the U.S. economy generally has recovered well, rates of inflation and food insecurity in the U.S. have risen, while ongoing disruptions to the supply chain and other pandemic-related issues have continued [11,17].

All the factors mentioned above can impact school meal operations and students’ and families’ experiences with USMs, highlighting the importance of continuing to evaluate the implementation of this program. Moreover, lessons learned from California’s USM evaluation can inform policy in other states, nationally and internationally. This study assessed the challenges and facilitators that Californian school food authorities (SFAs) faced while continuing to offer USMs under the state’s policy during SY 2022–2023.

## 2. Materials and Methods

### 2.1. Participants and Recruitment

In March 2023, all Californian SFAs participating in the NSLP (*n* = 1317) were invited to complete an online survey sharing their perspectives about providing school meals during SY 2022–2023, when California’s USM policy went into effect. The California Department of Education sent the email invitation along with two reminder emails to elicit additional participation; the survey link was open for six weeks. Participation in the survey was voluntary, and participants were not incentivized to participate. 

**Figure 1:**
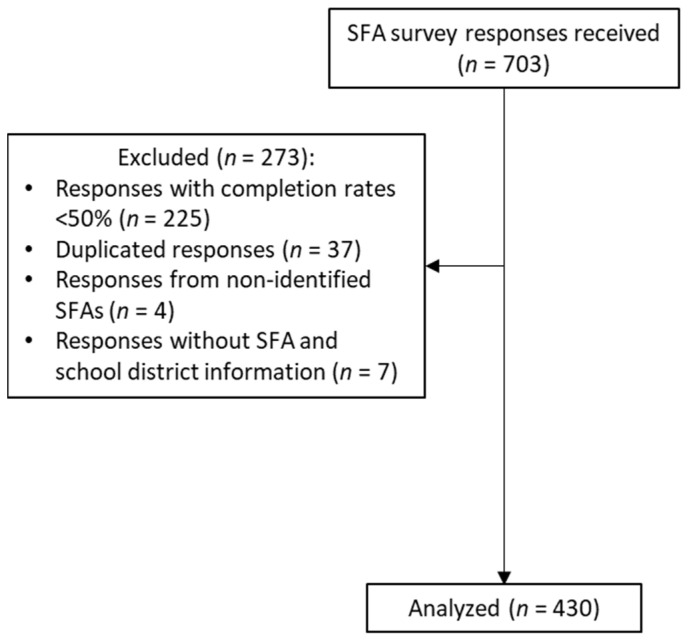
Participant flow chart for the 2023 California school food authority survey.

A total of 703 surveys were received: surveys with less than a 50% completion rate (*n* = 225), duplicated responses (*n* = 37), responses without information about the represented SFA (*n* = 4), and responses from SFAs not recognized by the California Department of Education or schools that were part of a larger SFA (*n* = 7) were excluded. The final analytical sample included 430 survey responses representing 32.6% of the SFAs in California (Figure 1).

### 2.2. Survey Instrument

Survey questions were adapted from previous instruments (*n* = 64) and covered multiple domains, including benefits and challenges in implementing the state’s USM policy, changes in meal offerings and foodservice operations, facilitators that helped USM policy implementation, and SFAs’ demographic characteristics (see Supplementary Materials) [10,18,19,20,21,22,23]. Most survey questions were multiple-choice with Likert scale response options. Subsequently, scaled variables were dichotomized for analytical purposes. The survey was programmed and administered in Qualtrics (Version March 2023, Provo, UT, USA) and took approximately 30–45 min to complete.

### 2.3. Stratification Variables

Analyses were stratified by select characteristics of SFAs, including the percentage of students eligible for FRPM and enrollment size, based on previous studies that showed that these characteristics can impact school meal operations and SFA experiences [10]. The percentage of students eligible for FRPM was reported by SFAs using the question: “Among the students within your entire SFA, approximately what percentage of students are eligible for FRPM based on meal applications, alternative income forms, direct certification, community eligibility, etc.?”. Answer options included: “<10%”, “10–24%”, “25–39%”, “40–59%”, “60–74%”, and “≥75%”. For the analysis by FRPM eligibility, SFAs were classified as having low FRPM eligibility (<40% of students eligible for FRPM) or high FRPM eligibility (≥40% of students eligible for FRPM). For the analysis by enrollment size, SFAs were categorized based on the total number of students in the SY 2022–2023 as small (<2500 students), medium (2500 to 9999 students), or large (≥10,000 students) [24]. Similar categories of student enrollment are used in the USDA’s professional standards [25].

### 2.4. Statistical Analysis

Categorical variables were described using frequencies and percentages. Differences by FRPM eligibility and enrollment size were examined using chi-squared tests. Pairwise comparisons between the enrollment size categories used the Bonferroni correction to account for multiple comparisons (Bonferroni = 0.025). Stata was used to conduct all the statistical analyses (StataCorp. 2023. Stata Statistical Software: Release 18. College Station, TX, USA: StataCorp LLC).

## 3. Results

### 3.1. Sample Characteristics

Table 1 shows the characteristics of the survey respondents and their SFAs. Most respondents were foodservice directors (63.9%), had been in their job for five years or more (52.4%), and represented small SFAs (54.9%). Most respondents represented SFAs with more than 40% of students eligible for FRPM (68.9%) and SFAs in urban areas (64.0%). Moreover, most of the represented SFAs began participation in CEP or Provisions 2 or 3 in SY 2019–2020 or earlier (62.4%) and had all schools currently participating in CEP or Provisions 2 or 3 (57.0%).

### 3.2. Benefits of Implementing California’s USM Policy during the SY 2022–2023

**Figure 2:**
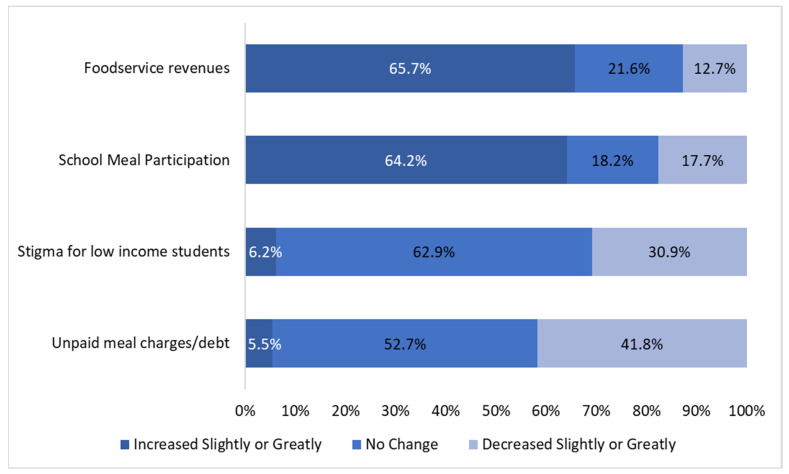
Benefits of implementing California’s USM policy during the SY 2022–2023, as reported by Californian school food authorities (*n* = 385, due to missingness of responses).

Most SFAs reported multiple benefits implementing California’s USM policy during the SY 2022–2023, including increases in foodservice revenues (65.7% of SFAs) and student meal participation (64.2%), as well as reductions in meal debt (41.8%) and student stigma (30.9%) (Figure 2). A sensitivity analysis comparing SFAs who began participation in CEP or Provision 2/3 in the school year 2019–2020 or earlier vs. SFAs newly implementing USMs showed that, for SFAs newly implementing USMs, school meal participation increased more (*p* < 0.01) and that student stigma and unpaid meal debt decreased more (*p* < 0.01 and *p* = 0.03, respectively) (Supplementary Table S1).

Stratified analyses by FRPM eligibility showed that, compared to SFAs with high FRPM eligibility, SFAs with low FRPM eligibility more often reported increases in student meal participation (76.7% vs. 58.8%, respectively) and reductions in stigma for low-income students (40.8% vs. 26.7%, respectively) (*p* < 0.05) (Table 2).

Stratified analyses by enrollment size showed that, compared with small SFAs, medium and large SFAs more often reported increases in foodservice revenues (77.3% and 80.9% vs. 54.6%, respectively) and reductions in stigma for low-income students (37.3% and 44.1% vs. 23.2%, respectively) (Table 2). Large SFAs reported reductions in unpaid meal debt more often than small SFAs (54.4% vs. 35.8%, respectively; *p* = 0.01).

### 3.3. Changes in Meal Offerings and Foodservice Operations Reported in Implementing California’s USM Policy in SY 2022–2023

**Figure 3:**
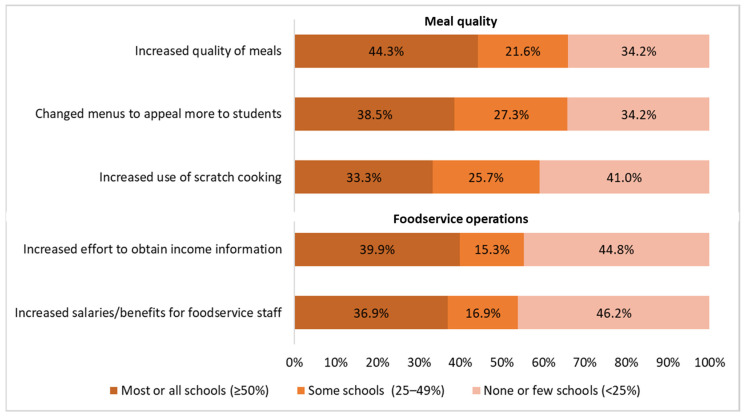
Changes in meal offerings and foodservice operations reported by Californian school food authorities in response to the state’s universal school meals policy during the SY 2022–2023 (*n* = 366, due to missingness of responses).

The most common changes in meal offerings and foodservice operations reported by SFAs included improving meal quality (44.3% of SFAs reported this change in at least half of their schools), increasing efforts to obtain income information from families (39.9%), adapting menus to appeal to different groups of students (38.5%), increasing salaries/benefits for foodservice staff (36.9%), and increasing the use of scratch/modified scratch cooking (33.3%) (Figure 3).

Stratified analyses by FRPM eligibility showed that, compared with SFAs with high FRPM eligibility, SFAs with low FRPM eligibility more often reported increases in the salaries of and benefits for foodservice staff (43.9% vs. 33.7%, respectively; *p* = 0.02) and efforts to obtain income information from families (50.0% vs. 34.9%, respectively; *p* < 0.01) (Table 3).

Stratified analyses by enrollment size showed that, compared with small SFAs, medium and large SFAs more often reported increases in meal quality (49.0% and 56.3% vs. 37.9%, respectively) and salaries/benefits for foodservice staff (50.0% and 48.4% vs. 26.3%, respectively) (*p* < 0.025) (Table 3). Medium SFAs more often reported an increased use of scratch cooking (36.5% vs. 30.8%, respectively; *p* < 0.01) and changing menus to appeal to different groups of students (44.2% vs. 32.8%, respectively; *p* = 0.02) than small SFAs.

### 3.4. Challenges While Implementing California’s USM Policy during the SY 2022–2023

**Figure 4:**
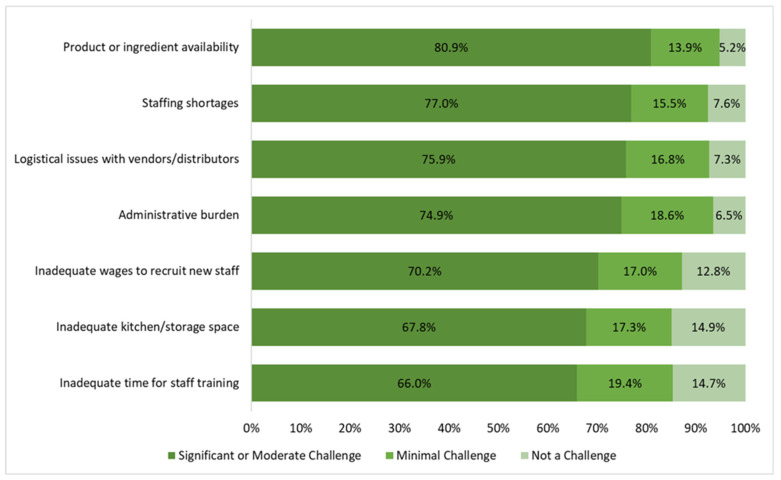
Challenges most commonly reported by Californian school food authorities related to providing school meals during the implementation of the state’s USM policy during SY 2022–2023 (*n* = 382, due to missingness of responses).

The most significant challenges reported by SFAs included product or ingredient availability (80.9% of SFAs reported this to be a significant or moderate challenge), staffing shortages (77.0%), logistical issues with vendors and distributors (75.9%), the administrative burden of the school meal program (74.9%), inadequate wages to recruit new staff (70.2%), inadequate kitchen facilities and/or storage space (67.8%), and inadequate time for staff training (66.0%) (Figure 4).

Stratified analyses by enrollment size showed that, compared to small SFAs, medium and large SFAs more often reported struggles with product or ingredient availability (88.2% and 89.7% vs. 74.0%, respectively), staffing shortages (92.7% % vs. 63.2%, respectively), logistical issues with vendors/distributors (85.5% and 91.2%, respectively), inadequate wages to recruit new staff (81.8% and 79.4% vs. 60.8%, respectively), inadequate kitchen facilities and/or storage space (76.4% and 80.9% vs. 58.8%, respectively), and inadequate time for staff training (71.8% and 77.9% vs. 58.8%, respectively) (*p* < 0.025) (Table 4). Similar challenges were reported by FRPM eligibility (*p* > 0.05). 

### 3.5. Facilitators to Implementing California’s USM Policy during SY 2022–2023

**Figure 5:**
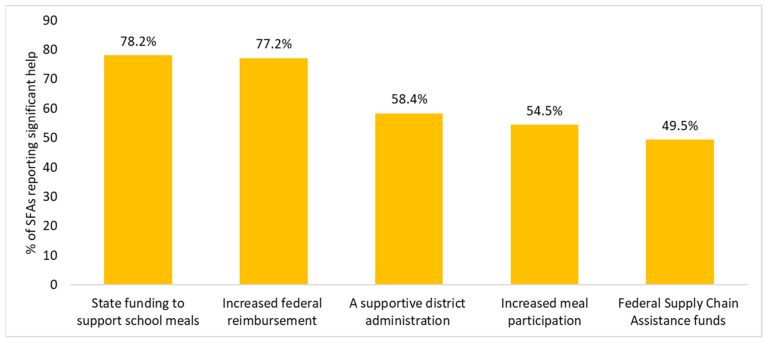
The most important factors identified by Californian school food authorities that helped them implement the state’s USM policy during SY 2022–2023 (*n* = 404, due to missingness of responses).

The top factors that helped support SFAs during the SY 2022–2023 include state funding to support school meals (78.2% of SFAs reported this to be significant help), increased federal school meal reimbursement rate (77.2%), a supportive district administration (58.4%), increased meal program participation (54.5%), and federal Supply Chain Assistance funds (49.5%) (Figure 5).

Some facilitators differed slightly for SFAs with high FRPM eligibility and larger SFAs (Table 5). For example, compared to SFAs with low FRPM eligibility, SFAs with high FRPM eligibility more often reported that federal Supply Chain Assistance funds (69.2% vs. 58.3%, respectively; *p* = 0.03) supported their implementation of USMs (Table 5). Compared to small SFAs, medium and large SFAs more often reported increased meal participation (85.3% and 87.3% vs. 72.8%, respectively), and federal Supply Chain Assistance funds supported their implementation of USMs (80.2% and 87.3% vs. 51.2%, respectively) (*p* < 0.025). Medium SFAs reported that state funding to support school meals supported their implementation of USMs more often than small SFAs (97.4% vs. 88.5%, respectively; *p* = 0.01). Compared with small SFAs, large SFAs more often reported that increased federal reimbursement (98.6% vs. 88.5%; *p* = 0.01) supported their implementation of USMs.

## 4. Discussion

California’s USM policy allowed schools to continue offering school meals free of charge to all students, regardless of their family income, during SY 2022–2023. SFAs reported that the first year implementing this policy was associated with multiple benefits, including increases in student meal participation and foodservice revenues, as well as reductions in unpaid meal charges and student stigma.

These findings, related to continuing to offer school meals free of charge to all California students, stand in contrast with national study findings from other states that reverted to charging for meals through the tiered eligibility system. No longer offering meals free for all students has been associated with a decline in school meal participation and an increase in stigma and meal debt during the SY 2022–2023 [27,28]. In fact, in one of these national studies, 80% of SFAs reported that meals no longer being free for all students were the primary driver of reduced student participation [27]. Similarly, a study examining student meal participation in states that maintained USM policies during the SY 2022–2023 (California, Maine, Massachusetts, Nevada, and Vermont) showed an increase in lunch participation compared to pre-pandemic levels, with school breakfast participation increasing in four out of the five states [7]. Previous studies evaluating the impact of USMs beyond the United States found that these programs in India and Sweden not only increased student participation but also improved their nutrition, health, and educational attainment [29,30,31].

In the present study, two-thirds of SFAs reported an increase in revenues, which may be partly explained by the increase in meal participation, higher reimbursement rates, and government investments to improve the quality and healthiness of school meals, which were reported by SFAs, as the top facilitators supporting the implementation of USMs. As a reference, the reimbursement rates for the NSLP and SBP during SY 2022–2023 were higher than those in SY 2021–2022, due to a 7.4% increase in the national average payment rates, plus a temporary additional 40 cents per lunch and 15 cents per breakfast due to the Keep Kids Fed Act of 2022 [32]. The increased support for school meal programs is critical, as previous studies found that SFAs needed additional resources, including facilities and equipment, staff, and higher meal reimbursement to offer healthier and more appealing school meals [33,34]. The impact of having funds to provide better-quality food to students is reflected in this study in the positive changes in meal offerings and foodservice operations reported by SFAs, including improvements in the quality of school meals, increased salaries and benefits for foodservice staff, and a shift towards more use of scratch or modified scratch cooking. Our findings highlight the early success of a state USM policy, in conjunction with additional investments in meal programs, in increasing students’ access to healthy school meals.

The reduction in unpaid meal charges could provide multiple benefits for school nutrition departments, schools, students, and families. The elimination of unpaid meal debt resulting from USM policies can save school nutrition departments time and resources that otherwise would be spent in debt-collection processes and can benefit schools by not having to use money from the general fund to cover unpaid meal debt or otherwise have to manage the negative balances of school meal programs. Students benefit by not being subjected to school cafeterias becoming a signal of family financial health and by not having school meals being associated with harmful debt-collection processes or penalties that cause stigma (e.g., lunch-shaming, the with-holding of official documents, and denying participation in student activities). Students benefit by having access to school meals, regardless of whether they can pay for them [35,36]. Finally, families can benefit from not having to worry about their child’s school meal program balance (saving them stress and money) and by not engaging with the school’s staff in debt-collection processes [6]. 

Despite the successes, SFAs faced multiple challenges implementing California’s USM policy during SY 2022–2023. The biggest challenges experienced by SFAs were not related to the USM policy but rather to other contextual factors, primarily supply chain and labor issues, including product or ingredient availability, staffing shortages, logistical issues with vendors and distributors, and administrative burdens. Similar results were reported in a recent national study that found SFAs most frequently reported increased costs, staffing shortages, and product shortages as challenges [27]. The similarities in the study findings suggest that these challenges are not unique to California but are likely reflective of broader national issues, possibly the lingering effects of the pandemic. Future studies should evaluate school foodservice challenges and facilitators once the supply chain issues and inflation rates ease. SFAs reported increases in administrative burden compared to the year prior, when the federal USM policy was in place. Unlike a federal USM policy, a state USM policy requires schools to collect students’ income eligibility information and identify students’ FRPM eligibility status at each meal to maximize the federal school meal reimbursements available under the tiered federal meal reimbursement system. The work of collecting meal application forms from parents in the context of meals being free of charge for all students is especially challenging because students can receive meals free of charge regardless of whether the forms have been completed, and parents may not know that the forms are still needed or understand why they should take the time to complete and return them now that the school meals are free for all students. A federal USM policy would reduce this administrative burden.

Other challenges reported by SFAs included inadequate wages to recruit new staff, inadequate kitchen facility and/or storage space, and inadequate time for staff training. Federal and state investments in recent years have aimed to address these issues by providing grants to upgrade school kitchens, train foodservice staff, and support schools to offer more fresh produce [14,16]. However, at the time of this evaluation, it was too early to assess the impact of those investments because most of the California SFAs awarded grants were still spending them or had purchased kitchen equipment but had not received it [37]. Future studies should evaluate the impact of federal and state investments on school foodservice operations and identify the SFAs needing more support to provide healthy and appealing school meals to students.

This study finds that meal participation increased more, and stigma declined more in SFAs with fewer students eligible for FRPM. Previous studies that evaluated the impact of offering USMs under the Community Eligibility Provision similarly found that students not eligible for FRPM experienced the greatest increases in participation [5,38]. In the present study, it is not known whether the SFAs with greater participation increases had more students eligible for FRPM participating once they were not exposing their family’s economic status by visiting the cafeteria or whether the increases were due to more students not formerly eligible for FRPM newly participating, or both. More meal participation and less stigma likely benefit students from many different economic and demographic backgrounds. SFAs with fewer students eligible for FRPM also more often reported increases in administrative burden and in the efforts to obtain income information from families. This may be because these SFAs may not be eligible to participate in the USDA’s provisions that allow schools and school districts in low-income areas to serve school meals at no cost to all enrolled students without collecting household applications and therefore make determining students’ income eligibility critical [39]. Finally, SFAs with fewer students eligible for FRPM more often reported increased salaries and benefits for foodservice staff. School foodservice jobs are often characterized by low pay and restricted work hours, conditions of employment that are better at many restaurants or other commercial foodservices [40]. SFAs might have to increase the salaries and benefits for foodservice staff to attract more workers. Addressing labor issues within school meal programs may be critical to their success.

Stratified analyses by enrollment size showed differences in how SFAs experienced the implementation of USMs. Larger SFAs reported experiencing more challenges implementing USMs than small SFAs, especially staffing shortages, which were 30 percentage points higher in larger SFAs. Similar results were reported in a national study where small SFAs (<1000 students) were marginally less likely than their counterparts to experience challenges [27]. However, our findings indicate that medium and large SFAs encountered more difficulties yet reported greater increases in foodservice revenues, meal quality, and salaries and benefits for foodservice staff. This could be attributed to economies of scale, where per-meal production costs decrease when more school meals are produced, generating more revenue that can be used to improve meal quality and foodservice staff salaries and benefits. Previous studies have shown that increases in student participation in school meal programs reduce per-meal production costs without reducing the nutritional quality of the meals [41,42,43]. Similar results were reported in previous national studies, wherein offering USMs through CEP was associated with lower meal costs among medium and large schools (≥500 students) but not among small schools (<500 students), and wherein small SFAs (<1000 students) were less likely to operate at a surplus or break even than larger SFAs [27,42]. These findings can inform policy and other supports needed by highlighting the need to provide the resources and infrastructure that medium and large SFAs need to serve school meals to a large body of students and provide the financial support that small SFAs require to compensate for their smaller economies of scale.

The strengths of this study include a sample representative of SFAs in the state of California based on FRPM eligibility (68.9% of SFAs in this study have ≥40% of students eligible for FRPM vs. 68.6% of schools in the state) [24]. However, the study sample did not necessarily represent SFAs in the state regarding enrollment size (54.9% of SFAs in this study were small vs. 66.7% in the state [information provided by the California Department of Education]). Another limitation is that we only received survey responses from a third of the SFAs in California, raising the risk of selection bias. It is possible that SFAs who had more or fewer challenges to report were more likely to volunteer to complete the survey. Another limitation is that all data were collected via the self-reports of food service directors. Future studies should capture experiences of stigma and perceptions of meal quality from students’ points of view.

## 5. Conclusions

Californian school food authorities found that the first year of implementation of the state’s USM policy during the 2022–2023 school year had multiple benefits, including increased meal participation, reduced meal debt, and less stigma associated with school meals. Additionally, SFAs report having improved meal quality and increased staff salaries. They reported ongoing challenges with broader contextual issues, including supply chain and staffing challenges, as well as inflation. Findings also suggest that targeted support for schools of various sizes and demographic characteristics may be important. Overall, the USM program appears to successfully increase student access to healthy meals and reduce stigma. Continued state and federal government support can help overcome operational challenges and ensure schools provide high-quality meals to optimally nourish students.

## Figures and Tables

**Table 1:** Characteristics of survey respondents and their Californian school food authorities (*n* = 431) ^1^.

**Title**	***n***	**%**
School Nutrition Director/Foodservice Director	274	63.9
School Nutrition Supervisor/Manager/Coordinator	97	22.6
Other	58	13.5
**Years in the role at SFA**	***n***	**%**
Less than 1 year	58	13.5
1–4 years	147	34.2
5–9 years	111	25.8
10 or more years	114	26.6
**Percentage of schools participating in CEP or Provision 2/3**	***n***	**%**
None	118	27.6
1–24% of schools	22	5.1
25–74% of schools	27	6.3
75–99% of schools	17	4.0
All schools	244	57.0
**The first year of participation in CEP or Provision 2 or 3, among those participating in one of those**	***n***	**%**
School year 2019–2020 or earlier	192	64.2
In the school year 2022–2023	107	35.8
**Enrollment size**	***n***	**%**
Small (2499 or fewer students)	236	54.9
Medium (2500–9999 students)	120	27.9
Large (10,000 or more students)	74	17.2
**Free and reduced-price meal (FRPM) eligibility**	***n***	**%**
Low FRPM eligibility (less than 40% of students)	132	31.1
High FRPM eligibility (40% or more of students)	292	68.9
**Urbanicity ^2^**	***n***	**%**
Urban	275	64.0
Not urban	155	36.1

^1^ Sample size varies for some questions due to missing survey responses. ^2^ Urbanicity was determined using the reported zip code and based on the 2010 USDA rural–urban commuting area (RUCA) codes, and it was dichotomized into urban (RUCA primary code = 1) and non-urban (RUCA primary codes = 2–10) [26]. SFA: school food authority; CEP: Community Eligibility Provision.

**Table 2:** Benefits of implementing California’s USM policy during the SY 2022–2023, as reported by Californian school food authorities, stratified by FRPM eligibility and enrollment size.

Change	FRPM Eligibility ^1^					Enrollment Size ^2^						
	Low (*n* = 120)		High (*n* = 262)		*p*-Value	Small (*n* = 207)		Medium (*n* = 110)		Large (*n* = 68)		*p*-Value
	*n*	%	*n*	%		*n*	%	*n*	%	*n*	%	
Changes that most reported as having increased ^3^												
Foodservice revenues	76	63.3	176	67.2	0.46	113	54.6	85	77.3	55	80.9	0.0001 ^a,b^
School meal participation	92	76.7	154	58.8	0.001	129	62.3	72	65.5	46	67.7	0.69
Changes that most reported as having decreased ^4^												
Stigma for low-income students	49	40.8	70	26.7	0.01	48	23.2	41	37.3	30	44.1	0.001 ^a,b^
Unpaid meal charges/debt	52	43.3	107	40.8	0.65	74	35.8	50	45.5	37	54.4	0.02 ^b^

^1^ Free or reduced-price meal (FRPM) eligibility was defined as SFAs with low FRPM eligibility (40% or fewer FRPM students) vs. high FRPM eligibility (SFAs with more than 40% FRPM students) in the SY 2022–2023. ^2^ Enrollment size was defined as small (≤2499 students), medium (2500 to 9999 students), and large (≥10,000 students). *p*-values for size represent the overall effect of enrollment size. The statistical significance for pairwise comparisons is indicated as follows: ^a^ difference between medium and small SFAs; ^b^ difference between large and small SFAs; no significant differences were observed between medium and large SFAs. ^3^ Frequencies representing SFAs that identified the changes as having increased slightly or greatly. Other answer options were: “no change”, “decreased slightly”, and “decreased greatly”. ^4^ Frequencies representing SFAs that identified the changes as having decreased slightly or greatly; other answer options were: “no effect”, “increased slightly”, and “increased greatly”.

**Table 3:** Changes in meal offerings and foodservice operations reported by Californian school food authorities implementing the state’s USM policy during the SY 2022–2023, stratified by FRPM eligibility and enrollment size.

Change	FRPM Eligibility ^1^					Enrollment Size ^2^						
	Low (*n* = 114)		High (*n* = 249)		*p*-Value	Small (*n* = 198)		Medium (*n* = 104)		Large (*n* = 64)		*p*-Value
	*n*	%	*n*	%		*n*	%	*n*	%	*n*	%	
**Meal quality ^3^**												
Increased quality of meals	54	47.4	108	43.4	0.17	75	37.9	51	49.0	36	56.3	0.001 ^a,b^
Changed menus to appeal to more students	46	40.4	95	38.2	0.85	65	32.8	46	44.2	30	46.9	0.03 ^a^
Increased use of scratch cooking	40	35.1	82	32.9	0.35	61	30.8	38	36.5	23	35.9	0.001 ^a^
**Foodservice operations ^3^**												
Increased effort to obtain income information	57	50.0	87	34.9	0.0001	76	38.4	51	49.0	19	29.7	0.09
Increased salaries/benefits for foodservice staff	50	43.9	84	33.7	0.02	52	26.3	52	50.0	31	48.4	0.0001 ^a,b^

^1^ Free or reduced-price meal (FRPM) eligibility was defined as SFAs with low FRPM eligibility (40% or fewer FRPM students) vs. high FRPM eligibility (SFAs with more than 40% FRPM students) in the SY 2022–2023. ^2^ Enrollment size was defined as small (≤2499 students), medium (2500 to 9999 students), and large (≥10,000 students). *p*-values for size represent the overall effect of enrollment size. The statistical significance for pairwise comparisons is indicated as follows: ^a^ difference between medium and small SFAs; ^b^ difference between large and small SFAs; no significant differences were observed between medium and large SFAs. ^3^ Frequencies representing SFAs that identified the changes in most or all schools (≥50%). Other answer options were: “some schools (25–49%)” and “none or few schools (<25%)”.

**Table 4:** Challenges reported by Californian school food authorities related to providing school meals during the implementation of the state’s USM policy during the SY 2022–2023, stratified by FRPM eligibility and enrollment size.

Challenge ^1^	FRPM Eligibility ^2^					Enrollment Size ^3^						
	Low (*n* = 118)		High (*n* = 260)		*p*-Value	Small (*n* = 204)		Medium (*n* = 110)		Large (*n* = 68)		*p*-Value
	*n*	%	*n*	%		*n*	%	*n*	%	*n*	%	
Product or ingredient availability	96	81.4	211	81.2	0.96	151	74.0	97	88.2	61	89.7	0.001 ^a,b^
Staffing shortages	93	78.8	197	75.8	0.52	129	63.2	102	92.7	68	92.7	0.0001 ^a,b^
Logistical issues with vendors/distributors	87	73.7	202	77.7	0.40	134	65.7	94	85.5	62	91.2	0.0001 ^a,b^
Paperwork/administrative burden of school meal program	94	79.7	189	72.7	0.15	146	71.6	85	77.3	55	80.9	0.24
Inadequate wages to recruit new staff	87	73.7	178	68.5	0.30	124	60.8	90	81.8	54	79.4	0.0001 ^a,b^
Inadequate kitchen facilities and/or storage space	86	72.9	171	65.8	0.17	120	58.8	84	76.4	55	80.9	0.0001 ^a,b^
Inadequate time for staff training	82	69.5	168	64.6	0.35	120	58.8	79	71.8	53	77.9	0.01 ^a,b^

^1^ Frequencies representing SFAs that identified the challenges as moderate or significant; other answer options were: “minimum challenge” and “not a challenge”. ^2^ Free or reduced-price meal (FRPM) eligibility was defined as SFAs with low FRPM eligibility (40% or fewer FRPM students) vs. high FRPM eligibility (SFAs with more than 40% FRPM students) in the SY 2022–2023. ^3^ Enrollment size was defined as small (≤2499 students), medium (2500 to 9999 students), and large (≥10,000 students). *p*-values for size represent the overall effect of enrollment size. The statistical significance for pairwise comparisons is indicated as follows: ^a^ difference between medium and small SFAs; ^b^ difference between large and small SFAs; no significant differences were observed between medium and large SFAs.

**Table 5:** Factors identified by Californian school food authorities that helped them implement the state’s USM policy during SY 2022–2023, stratified by FRPM eligibility and enrollment size.

Facilitators ^1^	FRPM Eligibility ^2^					Enrollment Size ^3^						
	Low (*n* = 127)		High (*n* = 273)		*p*-Value	Small (*n* = 217)		Medium (*n* = 116)		Large (*n* = 71)		*p*-Value
	*n*	%	*n*	%		*n*	%	n	%	*n*	%	
State funding to support school meals	118	92.9	253	92.7	0.93	192	88.5	113	97.4	69	97.2	0.003 ^a^
Increased federal reimbursement	113	89.0	256	93.8	0.10	192	88.5	110	94.8	70	98.6	0.01 ^b^
A supportive district administration	99	78.0	223	81.7	0.38	168	77.4	100	86.2	58	81.7	0.15
Increased meal participation	107	84.3	210	76.9	0.09	158	72.8	99	85.3	62	87.3	0.01 ^a,b^
Federal Supply Chain Assistance funds	74	58.3	189	69.2	0.03	111	51.2	93	80.2	62	87.3	0.0001 ^a,b^

^1^ Frequencies representing SFAs that identified the facilitators as significant or moderate help; other answer options were: “minimal help” and “not applicable”. ^2^ Free or reduced-price meal (FRPM) eligibility was defined as SFAs with low FRPM eligibility (40% or fewer FRPM students) vs. high FRPM eligibility (SFAs with more than 40% FRPM students) in the SY 2022–2023. ^3^ Enrollment size was defined as small (≤2499 students), medium (2500 to 9999 students), and large (≥10,000 students). *p*-values for size represent the overall effect of enrollment size. The statistical significance for pairwise comparisons is indicated as follows: ^a^ difference between medium and small SFAs; ^b^ difference between large and small SFAs; no significant differences were observed between medium and large SFAs.

## Data Availability

The original contributions presented in the study are included in the article, further inquiries can be directed to the corresponding authors.

## Supplementary Materials

The following supporting information can be downloaded at: https://www.mdpi.com/article/10.3390/nu16121812/s1, Table S1: Changes reported by Californian school food authorities while implementing California’s USM policy during the SY 2022–2023 among SFAS implementing USMs since 2019–2020 or earlier vs. those newly implementing USMs.
