# Supplementary material for: The Benefits and Challenges of Providing School Meals during the First Year of California’s Universal School Meal Policy as Reported by School Foodservice Professionals
Source: Nutrients. 2024 Jun 8;16(12):1812. doi: 10.3390/nu16121812 (PMC11206622; doi:10.3390/nu16121812)
Supplement: Supplementary file 1 [file nutrients-16-01812-s001.zip › Supplementary FSD Survey 2023.pdf]

2023 Online Statewide Survey of Foodservice Directors
Green text – California only

[Domain: Introduction]
INTRODUCTION Thank you for taking part in an evaluation of school meals. This survey will take approximately 30-45 minutes to complete. We will combine survey results from multiple school food authorities across the United States; **you and your schools will not be identified with your responses.**

Please answer the questions to the best of your knowledge. If you cannot answer a question, you can skip it, but we hope that you will answer all questions. If you need to stop before completing the survey, you can return and continue where you left off at any time before April 1st. Please ensure there is only one survey entry per school food authority. If you have any questions, please contact our research team at SchoolMealStudy@ucanr.edu

1. **In which STATE is your school food authority located?**
   a. Arizona ***[Control – hybrid as reduced price students are free]***
   b. California ***[UFSM]***
   c. Colorado ***[UFSM]***
   d. Illinois ***[Control]***
   e. Maine ***[UFSM]***
   f. Massachusetts ***[UFSM]***
   g. New Jersey ***[Control – hybrid as UFSM expanded to majority of schools but not all]***
   h. New Hampshire ***[Control]***
   i. Texas ***[Control]***
   j. Vermont ***[UFSM]***

2. ***[All states EXCEPT CA & TX]*** Over the next several months, we would like to interview some of you who take this survey. If we interview you, you will receive $25 and your responses will remain confidential. **May we contact you for a brief interview to learn more about your opinions of school meals?** (Interviews can be scheduled at a day and time that you choose).
   Yes No

3. ***[CA & TX ONLY]*** Over the next several months, we would like to interview some of you who take this survey. Your responses will remain confidential. **May we contact you for a brief interview to learn more about your opinions of school meals?** (Interviews can be scheduled at a day and time that you choose). Yes No

   3a. ***[CA ONLY]*** Next school year (2023-24) we will be working to understand the experiences of a small number of SFAs that have been engaged in, or are planning, new efforts to increase student participation in school breakfast and/or lunch. We will be asking a set of districts to join an evaluation of these efforts. The evaluation will involve collecting more detailed information about these efforts and surveying parents and students. **Would you like to learn more and be considered for this evaluation?**
      Yes No

(if Yes to Q2 or 3 or 4b above)
4. **Your email address:** ____

We will use this email address to contact you to schedule an interview. Your email will not be shared with anyone.
Please enter your email address a second time: ________________________________

5. **Your phone number:** ____
We will ONLY use this as a backup to contact you about scheduling an interview. Your phone number will not be shared with anyone.
Please enter your phone number a second time: ________________________________

[Domain: district foodservice director]

6. **Your Name:** First, Last *(In case we need to ask any questions about your responses)*

7. **Which of the following most appropriately describes your title(s)?** (*mark all that apply)*
   a. School Nutrition Director/Foodservice Director
   b. School Nutrition Supervisor/Manager/Coordinator
   c. Other *(write in)*

8. **How many years have you worked in this leadership role at your district?**
   a. Less than 1 year
   b. 1-4 years
   c. 5-9 years
   d. 10-14 years
   e. 15 or more years

[Domain: district demographics]

9. **Full District Name** *(please **do not use abbreviations**; if you provide school nutrition services to multiple districts, please provide your **primary district**)*

10. ***[ALL STATES EXCEPT CA]*** **Local Education Agency (LEA) ID (if known):** **_____________________________**

11. ***[CA ONLY]*** **Your District's assigned Child Nutrition and Information Payment System (CNIPS) Number** ___________________*(for accessing demographic school data so that you do not have to report this on the survey)* ***[CREATE DATA ENTRY CHECK FOR CORRECT NUMBER OF NUMERALS]***

12. **District Address** *(where you receive work mail)***:**
   a. Street
   b. City
   c. Zip

13. **Currently, how many districts are in your school food authority (SFA)**?
   a. 1
   b. 2
   c. 3
   d. 4
   e. 5+

14. **What types of schools are included in your SFA? Please select all that apply.** ***[USE TO CREATE***

SKIP PATTERNS FOR QUESTIONS ASKED by SCHOOL TYPE]

a. Elementary (e.g., grades K-5) Yes No
b. Middle or Junior high (e.g., grades 6-8) Yes No
c. High (e.g., grades 9-12) Yes No
   i. *[If Yes]* Do any high schools in your SFA have open campuses (some or all students can leave campus to purchase lunch)? Yes- All Yes- Some No Not Sure
d. K-8 and/or K-12 schools Yes No

15a. *[UFSM STATES ONLY]* What percent of schools in your SFA currently provide free school meals to all students specifically through CEP or Provision 2/3 (do not count schools implementing a state policy on Universal Free School Meals)?

a. None *[skip next question]*
b. 1% - 24% of schools
c. 25% - 74% of schools
d. 75% - 99% of schools
e. All schools

15b. *[CONTROL STATES ONLY]* What percent of schools in your SFA currently provide free school meals to all students specifically through CEP, Provision 2, or Provision 3 (For schools in New Jersey ONLY, please include expanded coverage for free school meals by the state)?

a. None *[skip next question]*
b. 1% - 24% of schools
c. 25% - 74% of schools
d. 75% - 99% of schools
e. All schools

[If answered there are some CEP schools in question 15a/15b]

***16.* When did the first schools in your SFA begin participating in CEP or Provision 2 or 3 for school lunch?**

a. School Year (SY) 2019 -20 or earlier
b. In SY 2022-23

***17. [CONTROL STATES ONLY]* Are free meals available to all students in your SFA through district, county, or private funding** (i.e., NOT including CEP or Provision 2 or 3)

a. Yes
b. No

***18.* Among the students within your entire SFA, approximately what percentage of students are eligible for free or reduced priced meals based on meal applications, alternative income forms, direct certification, community eligibility, etc.?**

a. <10%
b. 10-24%
c. 25-39%
d. 40-59%
e. 60-74%
f. >75%

[Domain: Time to Eat]

19. **What is the current amount of time scheduled for students' lunch period** (time to walk to the cafeteria, wait in line, and eat lunch [excluding time for recess]) ***[Coded to ONLY show school types based on response to Q14]***

- i. For Elementary school(s) [*insert choices a-g from below*]
  1. Do you think this is adequate and reasonable (taking into account the time to walk to the cafeteria, wait in line, and eat lunch [excluding time for recess])?
     a. If NO: What would be an adequate and reasonable amount of time scheduled for a lunch period (time to walk to the cafeteria, wait in line, and eat their lunch [excluding time for recess]) [*insert choices a-g from below*]
- ii. For Middle school(s) *[insert choices a-g from below*]
  1. Do you think this is adequate and reasonable?
     a. If NO: What would be an adequate and reasonable amount of time scheduled for a lunch period (time to walk to the cafeteria, wait in line, and eat lunch). [*insert choices a-g from below*]
- iii. For High school(s) [*insert choices a-g from below*]
  1. Do you think this is adequate and reasonable?
     a. If NO: What would be an adequate and reasonable amount of time scheduled for a lunch period (time to walk to the cafeteria, wait in line, and eat their lunch). [*insert choices a-g from below*]
- iv. For K-8/K-12 school(s) [*insert choices a-g from below*]
  1. Do you think this is adequate and reasonable?
     a. If NO: What would be an adequate and reasonable amount of time scheduled for a lunch period (time to walk to the cafeteria, wait in line, and eat their lunch). [*insert choices a-g from below*]

Choices:

a) Less than 15 minutes
b) 15-19 minutes
c) 20-24 minutes
d) 25-29 minutes
e) 30-34 minutes
f) 35-40 minutes
g) More than 40 minutes

[Domain: meal preparation & procurement]

20. **How are reimbursable school meals for your schools prepared?** (*mark all that apply)*

a. Prepared by a district nutrition services department/central kitchen
b. Prepared at school sites (do not count if schools mostly reheat and serve)
c. Another school district vends the meals
d. A foodservice management company or for-profit company that vends the meals
e. Other *(write in)*

21. **Does your SFA participate with other SFAs or another organization in a food-purchasing cooperative**?

a. Yes
b. No

22. **<u>In a typical week</u>, how many days at a <u>typical school</u> does your SFA use any of the following for reimbursable meals?** *Please select a response for each item.*

	No days per week	1 day per week	2 days per week	3 days per week	4 days per week	5 days per week
a. Convenience preparation (pre-portioned, heat and serve items, e.g., frozen burrito)						
b. Minimal preparation (primarily involving assembling and portioning, e.g., chef's salad made with pre-cut and pre-cooked ingredients)						
c. School-made/scratch or modified scratch preparation (use of minimally processed foods, some degree of ingredient preparation and cooking when needed, e.g., spaghetti with scratch-prepared sauce)						

22a. [If answer to 22c is one or more days] **During the current school year, what percent of all reimbursable entrées are freshly prepared onsite (scratch or modified scratch)?**

a. <10%
b. 10-24%
c. 25-39%
d. 40-59%
e. 60-74%
f. >75%
g. Don't know

23. **During the current school year, what is your best estimate of the percentage "locally grown or produced" foods purchased in your district (please consider "locally grown or produced" the same as you would on the farm to school census).**

a. <10%
b. 10-24%
c. 25-39%
d. 40-59%
e. 60-74%
f. >75%
g. Don't know

24a. *[UFSM STATES ONLY]* **Has your state's Universal Free School Meals policy impacted your SFA's ability to serve locally grown or produced foods?**

a. Made it <u>easier</u> to serve locally grown or produced foods

b. Made it harder to serve locally grown or produced foods
c. Not changed my ability to serve locally grown or produced foods

24b. *[CONTROL STATES ONLY]* Has discontinuing federal Universal Free School Meals impacted your SFA's ability to serve locally grown or produced foods?

a. Made it easier to serve locally grown or produced foods
b. Made it harder to serve locally grown or produced foods
c. Not changed my ability to serve locally grown or produced foods

24c. *[All STATES]* What factors (if any) have helped your SFA to serve more locally grown or produced foods? *(check all that apply)*

a. State meal reimbursement funds
b. State grant funds
c. Federal grant funds
d. Additional funding but not sure of the source
e. Support and technical assistance from a non-profit organization
f. We are not serving locally grown or produced foods

1. In a typical week, how many days at a typical school do you serve any organic foods?

a. No days
b. 1 day
c. 2 days
d. 3 days
e. 4 days
f. 5 days
g. Don't know

[Domain: meal service methods]

26. **Do you implement the following strategies for the reimbursable school BREAKFAST in any of your elementary, middle, high or K-8/K-12 schools?**
Please select a response for each item. ***[Coded to ONLY show school types based on response to Q14]***

	School type			
	**Elementary schools**	**Middle schools**	**High schools**	**K-8/K-12 schools**
a. Breakfast in the cafeteria before the bell	Yes- All<br>Yes- Some<br>No	Yes- All<br>Yes- Some<br>No	Yes- All<br>Yes- Some<br>No	Yes- All<br>Yes- Some<br>No

	N/A	N/A	N/A	N/A
b. "Grab and go" option(s) (packaged, carry-away or other non-congregate option)	Yes- All<br>Yes- Some<br>No<br>N/A	Yes- All<br>Yes- Some<br>No<br>N/A	Yes- All<br>Yes- Some<br>No<br>N/A	Yes- All<br>Yes- Some<br>No<br>N/A
c. Breakfast after the bell in the classroom or other instructional location (counted as instructional minutes)	Yes- All<br>Yes- Some<br>No<br>N/A	Yes- All<br>Yes- Some<br>No<br>N/A	Yes- All<br>Yes- Some<br>No<br>N/A	Yes- All<br>Yes- Some<br>No<br>N/A
d. "Second chance" breakfast (available after school begins or during first period but not served in the classroom)	Yes- All<br>Yes- Some<br>No<br>N/A	Yes- All<br>Yes- Some<br>No<br>N/A	Yes- All<br>Yes- Some<br>No<br>N/A	Yes- All<br>Yes- Some<br>No<br>N/A
e. Other strategies (e.g., changes in bus schedules or times for breakfast, leftover breakfast foods made available later in the day) *(write in)*	Yes- All<br>Yes- Some<br>No<br>N/A	Yes- All<br>Yes- Some<br>No<br>N/A	Yes- All<br>Yes- Some<br>No<br>N/A	Yes- All<br>Yes- Some<br>No<br>N/A

***27.* Do you implement the following strategies for the reimbursable school LUNCH in any of your elementary, middle, high or K-8/K-12 schools?**
Please select a response for each item. ***[Coded to ONLY show school types based on response to Q14]***

	**School type**			
	**Elementary schools**	**Middle schools**	**High schools**	**K-8/K-12 schools**
a. Recess before lunch	Yes- All<br>Yes- Some<br>No<br>N/A	Yes- All<br>Yes- Some<br>No<br>N/A	Yes- All<br>Yes- Some<br>No<br>N/A	Yes- All<br>Yes- Some<br>No<br>N/A
b. "Grab and go" option(s) (packaged, carry-away or other non-congregate option)	Yes- All<br>Yes- Some<br>No<br>N/A	Yes- All<br>Yes- Some<br>No<br>N/A	Yes- All<br>Yes- Some<br>No<br>N/A	Yes- All<br>Yes- Some<br>No<br>N/A
c. "Offer-versus-serve" option (students are asked whether or not they want a particular meal item)	Yes- All<br>Yes- Some<br>No<br>N/A	Yes- All<br>Yes- Some<br>No<br>N/A	Yes- All<br>Yes- Some<br>No<br>N/A	Yes- All<br>Yes- Some<br>No<br>N/A

d. Option(s) for students to pre-order	Yes- All<br>Yes- Some<br>No<br>N/A	Yes- All<br>Yes- Some<br>No<br>N/A	Yes- All<br>Yes- Some<br>No<br>N/A	Yes- All<br>Yes- Some<br>No<br>N/A
e. More than one serving line or food station (include mobile carts, kiosks, service windows, salad bars or other self-serve bars)	Yes- All<br>Yes- Some<br>No<br>N/A	Yes- All<br>Yes- Some<br>No<br>N/A	Yes- All<br>Yes- Some<br>No<br>N/A	Yes- All<br>Yes- Some<br>No<br>N/A
f. All meal components are provided on every serving line or food station in the required minimum amounts	Yes- All<br>Yes- Some<br>No<br>N/A	Yes- All<br>Yes- Some<br>No<br>N/A	Yes- All<br>Yes- Some<br>No<br>N/A	Yes- All<br>Yes- Some<br>No<br>N/A
g. Students must visit multiple serving lines or food stations that together offer all required meal components (e.g., pasta station, fruit and vegetable bar, and milk cooler)	Yes- All<br>Yes- Some<br>No<br>N/A	Yes- All<br>Yes- Some<br>No<br>N/A	Yes- All<br>Yes- Some<br>No<br>N/A	Yes- All<br>Yes- Some<br>No<br>N/A

[Domain: barriers to meal participation]

28. **Thinking about the students who do NOT regularly eat the reimbursable school meals, how common do you think the following barriers are for the students you serve?** *Please select a response for each item.*

	**None or very few students (<25%)**	**Some students (25-49%)**	**Many or most Students (≥50%)**	**Don't know/ not sure**
a. Students do not like the taste of the food				
b. Students do not think the food is fresh				
c. Students or parents do not think the food is healthy				
d. Students prefer to eat a la carte options				
e. Students get tired of the options				
f. Foods do not meet students' cultural or non-medical dietary preferences				
g. Portions are not big enough / not enough food provided				
h. Students prefer to eat meals from home or elsewhere				
i. Students often skip meals (e.g., do not eat any breakfast or lunch)				
j. Students unable to get to school on time for breakfast				
k. Students don't have enough time to get and eat the lunch				
l. Lunch lines are too long				

m. Students or parents think only needy kids eat school meals and don't want to be thought of that way				
n. Students' friends don't eat the school meals				
o. ***[UFSM States ONLY]*** Students or parents don't understand that meals are free				
p. ***[Arizona ONLY]*** Students or parents don't understand that meals are free for students eligible for reduced price meals				
q. ***[Control States ONLY]*** The cost of school meals is too high for students who are not eligible for free or reduced-price meals				
r. Other *(write in)*				

29. **Some SFAs are working to increase student participation in school breakfast and/or school lunch. This may involve changing the meals or how they are served, promoting the meals, or other efforts. Are you doing (or plan to do) any of the following?**

	Currently doing	Planning to do	Not currently doing or planning to do
Social media outreach to parents			
Social media outreach to students			
Other parent communications – newsletters, emails, flyers			
New menu items			
Student taste testing			
Student feedback			
Parent feedback			
Breakfast after the bell			
Second chance breakfast			
Breakfast in the classroom			
More grab and go options			
Increased scratch cooking/modified scratch cooking			
Upgraded or redesigned serving area			
Upgraded or redesigned eating area			
More point of service stations			
Longer meal times			
Increased plant-based meal options			
Increased menu variety			
New salad bars			
Smarter Lunchroom practices			

Other *(write in)*			

[Domain: innovations and new funding]

30. **Across the following service functions, has equipment been purchased this school year (SY 2022-23 [include if you are planning or in the process of procuring for SY 2023-24]) for any kitchens in your SFA?** *Please select a response for each item.*

	**No**	**Yes, and we are already using it**	**Yes, it is ordered and will be arriving soon**	**Yes, it is ordered but delivery is delayed**	**Yes, but we haven't ordered it yet**
Receiving and storage (e.g., platforms and hand trucks, scales, or walk-in refrigerators/ freezers)					
Food preparation (e.g., slicers, food processors, utility carts, stainless steel work tables, or combi ovens)					
Holding and transportation (e.g., refrigerated or non-refrigerated trucks, hot holding mobile carts, or walk-in coolers [separate from receiving/storage refrigerators])					
Salad or fruit/vegetable bars					
Other meal service equipment (e.g., mobile milk coolers, steam table pans or serving portion utensils)					
Reusable instead of disposable service ware (e.g., washable plates, trays, eating utensils)					
Technology (e.g., point-of-service software, phone app technology)					

31. **Which of the following have helped support your SFA this school year (SY 2022-23)?** *Please select a response for each item.*

	**Significant Help**	**Moderate Help**	**Minimal Help**	**Not applicable**
Increased federal reimbursement rate/funding of school meals				
Availability of state funding to support school meals				
Availability of local funding to support school meals				
Availability of federal Supply Chain Assistance (SCA) funds				
Technical assistance (e.g., webinars) provided by the state				
Technical assistance (e.g., webinars) provided by nonprofit organizations				
A supportive district administration				
Increased meal program participation				
Political or public support or attention regarding school meals				
Other *(write in)*				

***32.* In the last 2 school years (SY 2021-22 and SY 2022-23) has your SFA been awarded any federal grants for the following?** *Please select a response for each item.*

	Yes, and already spent	Yes, but still spending	Pending and not yet received	No, applied but not awarded	No, we did not apply
New kitchen equipment					
Foodservice workforce training					
Increase procurement of local produce (e.g., farm to school)					
Other *(write in)*					

***33.* In the last 2 school years (SY 2021-22 and SY 2022-23) has your SFA been awarded any state or local grants for the following?** *Please select a response for each item.*

	Yes, and already spent	Yes, but still spending	Pending and not yet received	No, applied but not awarded	No, we did not apply or funding not available in my state
a) New kitchen equipment					
b) Foodservice workforce training					
c) Increase procurement of local produce (e.g., farm to school)					
d) Other *(write in)*					

[Domain: challenges]

***34.* In implementing school meals, how challenging are the following foodservice operations?** *Please select a response for each item.*

	Significant Challenge	Moderate Challenge	Minimal Challenge	Not a Challenge
Paperwork/administrative burden of school meal program				
Costs/financial sustainability of school meal programs				
Decrease in revenue from paid meals due to the capped paid meal reimbursement rate				
Decrease in revenue from competitive food and beverage sales managed by your SFA				
Product or ingredient availability				
Sourcing locally grown or produced items				
Meeting federal school meal nutrition standards				
Maintaining meal quality and variety				
Logistical issues with vendors/distributors				

Staffing shortages				
Inadequate wages to recruit new staff				
Inadequate time for staff training				
Inadequate kitchen equipment				
Inadequate kitchen facility and/or storage space				
Low breakfast participation				
High breakfast participation				
Low lunch participation				
High lunch participation				
State reimbursement process (*leave blank if not applicable*)				
Unpaid meal debt				
Other *(write in)*				

35. **How challenging are the following cafeteria operations?** *Please select a response for each item.*

	Significant Challenge	Moderate Challenge	Minimal Challenge	Not a Challenge
Long time in line for students to get meals/length of lines				
Not enough time for students to eat				
Inadequate meal service space				
Inadequate/crowded dining space				
Not enough kitchen preparation space				
Not enough refrigeration/freezer space				
Not enough points of service				

36. **How challenging are the following issues related to people or groups interested in school meals?** *Please select a response for each item.*

	Significant Challenge	Moderate Challenge	Minimal Challenge	Not a Challenge
Student and parent complaints				
Meeting student cultural/ethnic food preferences				
Meeting student needs for variety				
Meeting student food allergies/medical nutrition needs				
Obtaining income information from families (e.g., meal applications, household income forms, etc.)				
Stigma for students from families with low income				

Lack of support from district administration (school board, superintendent)				
Lack of support from school administration (principals/vice principals)				
Lack of support from nutrition services staff				
Lack of support from classroom teachers, school nurses, janitorial staff, and other school personnel				
Lack of support from school or district wellness committees				
[Massachusetts and Vermont ONLY] Lack of financial support from federal government or state for Universal Free School Meals beyond SY 2022-23				
[Control States ONLY] Lack of financial support from federal government or state for Universal Free School Meals in your state this school year (SY 2022-23)				
[Arizona ONLY] Lack of financial support from federal or state government for covering co-pay for reduced price meals beyond SY 2023-24				

37. **In implementing school meals in SY 2022-23, how much have the following CHANGED from last year (SY 2021-22).** *Please select a response for each item.*

	Decreased Greatly	Decreased Slightly	No Change	Increased Slightly	Increased Greatly
School Meal Participation					
Paperwork/administrative burden					
Stigma for students from families with low income					
Unpaid meal charges/debt					
Foodservice revenues					
Foodservice staffing challenges					
Number of student and parent complaints					
Number of student and parent compliments related to school meals					
Ability to meet student cultural/ethnic food preferences					
Ability to meet student food allergies/medical nutrition needs					
Ease of obtaining income information from families					
Degree of support from district administration (school board, superintendent)					
Degree of support from school administration (principals/vice principals)					
Degree of support from nutrition services staff					
Degree of support from classroom teachers, school nurses, janitorial staff, and other school personnel					
Degree of support from school or district wellness committees					

[Control States ONLY] Parent or student confusion regarding the cost of school meals					
Other *(write in)*					

[Domain: resource needs]

38. **Thinking ahead for next year (SY 2023-24), what additional technical assistance and/or trainings, if any, could your SFA benefit from?**
Please select a response for each item.

	**Not Needed**	**Needed a Little**	**Needed a Lot**
Financial management			
Working through staffing challenges			
Procurement			
Menu planning, meal counting and claiming			
Cooperative purchasing			
Cultural diversity in meal planning			
Meeting special dietary needs			
Communications and marketing to students and parents			
Making school meals more appealing to students			
Increasing school meal participation			
Getting more families to submit income information			
Direct certification with Medicaid			
Improving procurement efforts (e.g., addressing supply chain issues)			
Ways to better engage **students** in school foodservices			
Ways to better engage **parents** in school foodservices			
Scratch cooking			
Serving more plant-based meals			
Local sourcing			
Organic sourcing			
Other *(write in)*			

[Domain: a la carte]

39. **Does your SFA sell food or beverages on an à la carte basis during school breakfast or lunch** in any of your schools (NOT including milk)?

a. Yes
b. No

40. **How many schools in your SFA sell competitive foods during school hours (in vending machines, school stores, or à la carte)?** *Please select a response for each item.* ***[Coded to ONLY show school types based on response to Q14]***

	**No schools (0)**	**Few schools (<25%)**	**Some schools (25-49%)**	**Most schools (50-99%)**	**All schools (100%)**
Elementary					
Middle					
High					
K-8/K-12					

[Domain: finances]

41. **Is the meal reimbursement (federal, state, and local combined, if applicable) for <u>BREAKFAST</u> sufficient for your SFA to cover the full cost of producing meals (including food, labor, and supplies, including those related to the pandemic)?**
Yes No Don't serve breakfast Don't know/Not sure ***[skip Q43 if marked any but No]***

42. **Is the meal reimbursement (federal, state, and local combined, if applicable) for <u>LUNCH</u> sufficient for your SFA to cover the full cost of producing meals (including food, labor, and supplies, including those related to the pandemic)?**
Yes No Don't know/Not sure ***[skip to Q45 if marked any but No for both breakfast and lunch]***

43. **Approximately what percent of the full cost of producing a school meal does the reimbursement for <u>breakfast</u> cover?**

Less than 25%

25-50%

51-75%

76-99%

Don't know

44. **Approximately what percent of the full cost of producing a school meal does the reimbursement for <u>lunch</u> cover?**

a. Less than 25%
b. 25-50%
c. 51-75%
d. 76-99%

e. Don’t know

45. Is your school nutrition program included in the local school budget?

a. Yes
b. No ***[skip next question]***
c. Don’t know / not sure ***[skip next question]***

44a. *[If YES]* Approximately how much did you (or will you) receive this school year in local funds?

Less than $20,000

$20,000-$49,000

$50,000- $99,000

$100,000 or more

45a. Which of the following factors are impacting revenues for your SFA this school year (SY2022-23)? *(mark all that apply)* ***[Ask only if marked No to breakfast and/or lunch in Q41]***

d. Food costs	Yes No
e. Labor costs	Yes No
f. Supply costs	Yes No
g. Equipment costs	Yes No
h. Facility costs	Yes No
i. Storage costs	Yes No
j. Transportation costs	Yes No
k. Indirect costs (fringe benefits, accounting, payroll, purchasing, facilities managements and utilities)	Yes No
l. Increased meal program participation	Yes No
m. Decreased meal program participation	Yes No
n. Decrease in competitive food sales	Yes No
o. ***[Control states only]*** Unpaid student meal debt	Yes No
p. Don’t know / not sure	Yes No
q. Other *(write in)*	

45b. What **minimum per meal reimbursement rate** do you think is necessary for your SFA to provide meals that meet all federal nutrition standards **and** appeal to students? ***[INCLUDE CHECKS SO ASKED TO VERIFY IF ABOVE $10.00 or below $1.00]***

a. Breakfast $_._ _
b. Lunch $_._ _

45c. What **minimum per meal reimbursement rate** do you think is necessary for your SFA to provide meals that regularly include fresh, locally grownproduce? ***[INCLUDE CHECKS SO ASKED TO VERIFY IF ABOVE $10.00 or below $1.00]***

a. Breakfast $_._ _
b. Lunch $_._ _

[Domain: changes due to universal free school meals]

46a. [UFSM states ONLY] **In response to your state's Universal Free School Meals policy, have you made any of the following changes this school year (SY2022-23)?** *Please select a response for each item.*

	None or few schools (<25%)	Some schools (25-49%)	Most or all schools (≥50%)
Started a new breakfast or lunch service (where there hadn't been one before)			
Started a breakfast after the bell program			
Moved lunch periods earlier or later in the day			
Added more serving lines or food stations			
Reduced the number of serving lines or food stations			
Shortened lunch periods			
Lengthened lunch periods			
Hired more foodservice staff			
Increased salaries/benefits for foodservice staff			
Found new food supplier(s)			
Reduced use of scratch/modified scratch cooking			
Increased use of scratch/modified scratch cooking			
Reduced quality of meals			
Increased quality of meals			
Changed menus to appeal to different groups of students			
Increased price or promotion of competitive foods to generate revenue			
Ended sale of competitive foods			
Increased effort to get income information from families			
Decreased effort to get income information from families			
Other *(write in)*			

_46b._ *[Control states ONLY]* In response to de-implementing the federal Universal Free School Meals policy, have you made any of the following changes this school year (SY2022-23)? *Please select a response for each item.*

	**None or few schools (<25%)**	**Some schools (25-49%)**	**Most or all schools (≥50%)**
Started a new breakfast or lunch service (where there hadn't been one before)			
Moved lunch periods earlier or later in the day			
Added more serving lines or food stations			
Reduced the number of serving lines or food stations			
Shortened lunch period			
Lengthened lunch periods			
Reduced the number of foodservice staff			
Found new food supplier(s)			
Reduced use of scratch/modified scratch cooking			
Increased use of scratch/modified scratch cooking			
Reduced quality of meals			
Increased quality of meals			
Changed menus to appeal to different groups of students			
Increased price or promotion of competitive foods to generate revenue			
Ended sale of competitive foods			
Increased effort to get income information from families			
Used direct certification with Medicaid to certify kids for free or reduced priced meals			
Other *(write in)*			

[Domain: household income verification]

_47._ Which of the following methods is your SFA collecting to determine student eligibility for free or reduced-price meals? (*mark all that apply)* ***[SKIP IF ALL SCHOOLS ARE CEP/PROVISION 2/3]***

a. Meal applications Yes No
b. Alternative income forms Yes No
c. Direct certification Yes No
d. Other *(write in)* Yes No

[If mark yes to a and/or b above ask Q47a, b, c]

_47a_. Do you have an online (paperless) option for collecting meal applications and/or alternative income forms?

a. Yes
b. No

***47b*. What is your estimated return rate for meal applications?**
a. ___%
b. Don't know

***47c*. What is your estimated return rate for alternative income forms?**
a. ___%
b. Don't know

[If yes to q47 response c – direct certification – ask 47d]
***47d.* How frequently do you update your direct certification lists:**
a. At least once per month
b. About every other month (4-5 times per year)
c. About once every three months (2-3 times/year)
d. Once per year
e. Never—we do not use direct certification

48.* Compared to before the pandemic (SY 2019-20), how has the percent of families returning meal application or household income forms changed?** ***[SKIP IF ALL SCHOOLS ARE CEP/PROVISION 2/3 and If mark NO to Meal Applications or Household Income Forms in Q47]
a. It hasn't changed
b. More families return the forms now compared to before the pandemic (higher rate of return)
c. Fewer families return the forms now compared to before the pandemic (lower rate of return)
d. Don't know

49. ***[ONLY For schools that answered YES to Meal Applications or Household Income Forms in Q47]*** **This school year (SY 2022-23), how many times have you used the following methods to inform parents about completing school meal applications or household income forms?**
Please select a response for each item.

	Never	1-3 times	4-6 times	7+ times
Posted information on a website (count as frequently as your website is substantially updated)				
Sent email directly to parents				
Sent text messages to parents				
Called parents by phone/auto-dialer				

Posted information on social media				
Distributed hardcopy communications to be sent home (e.g., flier, posters, or newsletter)				
Distributed hardcopy communications at in-person events (such as at back-to-school night or parent-teacher meetings)				
Used the school's public address system for announcements				
Included with school registration packets				
Provided information in a language other than English				
Other *(write in)*				

49a. ***[Arizona ONLY]*** **Department of Education implemented a policy to eliminate co-pays for reduced price meals starting in January 2023. What strategies did schools in your FSA use to inform parents about this change?** Select all that apply.

a. Sent text messages to parents
b. Called parents by phone
c. Sent emails to parents
d. Sent fliers home
e. Posted information on social media
f. Updated school website
g. Other *(write in)*

50. ***[UFSM states ONLY]*** **If the current Universal Free School Meals policy were to end in your state, what effects, if any, do you think this would have on the following?** *Please select a response for each item.*

	Decrease Greatly	Decrease Slightly	No Effect	Increase Slightly	Increase Greatly
Student meal participation					
School meal quality					
Paperwork/administrative burden					
Stigma for students from families with low income					
Unpaid meal charges/debt					
Foodservice revenues					
Foodservice staffing challenges					
Parent or student confusion regarding the cost of school meals					
Other *(write in)*					

***50a. [Arizona ONLY]* Arizona Department of Education implemented a policy to eliminate co-pays for reduced price meals starting in January 2023. If this policy were to end, what effects, if any, do you think this would have on the following?** *Please select a response for each item.*

	Decrease Greatly	Decrease Slightly	No Effect	Increase Slightly	Increase Greatly
a. Student meal participation					
b. School meal quality					
c. Paperwork/administrative burden					
d. Stigma for students from families with low income					
e. Unpaid meal charges/debt					
f. Foodservice revenues					
g. Foodservice staffing challenges					
h. Parent or student confusion regarding the cost of school meals					
i. Other *(write in)*					

[Domain: USDA proposed rule changes]

***51.* The USDA has proposed updates to school meal nutrition standards to be phased in beginning SY 2025-26 in alignment with the 2020 Dietary Guidelines for Americans. How challenging or easy will the following changes be for your SFA?** *Please select a response for each item.*

	Very Challenging	Somewhat Challenging	A little Challenging	Neutral	A little Easy	Somewhat Easy	Very Easy
Limit added sugars in grain-based desserts and breakfast cereals							
Limit added sugars in yogurt and flavored milk							
Limit weekly added sugars to <10% of total calories							
Allow flavored milk (fat-free and low-fat) in high schools only							
Serve at least 80% of the weekly grains as whole grain							
Serve only whole grain-rich grains except for one day per week when enriched grains can be included							
Reduce sodium in school breakfasts from the current limit by 10% per year over two years							
Reduce sodium in school lunches from the current limit by 10% per year over three years							

[Domain: closing]

***52.* Please share any additional comments you have about the school meals programs.** *(write in)*

_53._ The study team is collaborating with policy makers, advocates, funders, technical assistance providers, and community groups interested in school meals. We will NOT share your data with anyone, unless you check this box that you would like us to.

May we share your data? (This will enable organizations to reach out to you regarding individualized support for your school meal program) Yes No

[Skip if CA or TX]

54. Q74 If you would you like to be entered in a raffle for a chance to win one of fifty $100 Amazon gift cards for taking this survey, please enter your email address below. We will ONLY use this email address to send the gift card for your participation. Your email address:

____

Thank you for sharing your information and expertise!
