# Supplementary material for: The Benefits and Challenges of Providing School Meals during the First Year of California’s Universal School Meal Policy as Reported by School Foodservice Professionals
Source: Nutrients. 2024 Jun 8;16(12):1812. doi: 10.3390/nu16121812 (PMC11206622; doi:10.3390/nu16121812)
Supplement: Supplementary file 1 [file nutrients-16-01812-s001.zip › Supplementary Table S1.pdf]

Supplementary Table S1. Benefits of implementing California's USM policy during the SY 2022-23 among California school food authorities implementing USM since 2019-20 or earlier vs. those newly implementing USM.

Change	SFAs implementing USM since 2019-20 or earlier (n=171)[1]		SFAs newly implementing USM (n=204)[2]		P-value
	n	%	n	%	
**Changes that most reported as having increased[3]**					
Foodservice revenues	117	68.4	131	64.2	0.39
School meal participation	94	55.0	146	71.6	0.001
**Changes that most reported as having decreased[4]**					
Stigma for low-income students	36	21.1	80	39.2	0.0001
Unpaid meal charges/debt	61	35.7	96	47.1	0.03

1. Includes SFAs who began participation in CEP or Provision 2/3 in the school year 2019 -20 or earlier.
2. Includes SFAs who began participation in CEP or Provision 2/3 in the school year 2022-23 or whose schools do not participate.
3. Frequencies representing SFAs that identified the changes as having increased slightly or greatly. Other answer options were: "no change," "decreased slightly," and "decreased greatly."
4. Frequencies representing SFAs that identified the changes as having decreased slightly or greatly; other answer options were: "no effect," "increased slightly," and "increased greatly".
